# Is there a gender effect in polycythemia vera?

**DOI:** 10.1007/s00277-020-04287-w

**Published:** 2020-10-02

**Authors:** Francesca Palandri, Barbara Mora, Naseema Gangat, Lucia Catani

**Affiliations:** 1 Azienda Ospedaliero-Universitaria di Bologna, Via Albertoni 15, Bologna, Italy; 2 Hematology, ASST Sette Laghi, Ospedale di Circolo, Varese, Italy; 3 Division of Hematology, Mayo Clinic, Rochester, MN USA

**Keywords:** Polycythemia vera, Gender, Therapy, Outcome

## Abstract

In recent times, there has been a growing interest in understanding the impact of gender on disease biology and clinical outcomes in Philadelphia-negative chronic myeloproliferative neoplasms. Among those, polycythemia vera (PV) is characterized by increased thrombotic risk, systemic symptoms, and overall reduced survival. Here, we aim to summarize data on whether and to what extent female sex can affect PV biology and outcome. To this end, we will discuss the latest acquisitions in terms of pathogenesis, diagnosis, epidemiology, clinical presentation and symptoms burden, thrombotic risk and related treatment strategies, and prognosis in female patients affected by PV.

## Introduction

Polycythemia vera (PV) is included among the Philadelphia-negative chronic myeloproliferative neoplasms (MPNs), together with essential thrombocythemia (ET) and primary myelofibrosis (PMF) [1]. PV and ET could evolve into post-PV or post-ET myelofibrosis (PPV/PET-MF), also known as secondary myelofibrosis (SMF) [2]. PV is more common than MF [3]. It is characterized by excessive red cell production and the release of pro-inflammatory cytokines [4]. These events are secondary to the hyper-activation of the JAK-STAT pathway, caused by mutations in *Janus kinase 2* (*JAK2*) gene [4]. Clinical phenotype is dominated by systemic symptoms and microvascular disturbances; in 30% of cases, splenomegaly may be detected [5, 6]. In the long term, what really affects outcome is the increased risk of thrombosis, evolution into PPV-MF, or into blast phase (BP) [7].

Gender-based discrepancies have been observed in terms of incidence, response to therapies, and prognosis in several solid and hematological cancers [8]. Sex differences may depend on multiple factors, including differentially activated genetic/molecular patterns, immune system function, sex hormones expression [9], and drug metabolism [10]. In solid cancers, as in acute leukemia, incidence, and mortality rates are higher in males. Overall mortality depends not only on multiple factors, including cardiovascular risk factors (which are generally more frequent in males), but also on genetic polymorphisms involved in facilitating carcinogenesis [11]. Besides, increased predisposition to leukemia in males could depend on the lack of estrogens [12]. The latter are supposed to inhibit the *Nuclear Factor kappa B* (NF-κB) pathway, which regulates the transcription of *Interferon Regulatory Factor 4* (IRF4) and, as a consequence, a correct differentiation of B and T cells [12]. Looking at the treatment effect, the anti-CD20 monoclonal antibody rituximab (used in several B lymphoproliferative disorders) seems to achieve better results in females [13, 14]. This is probably due to a faster drug clearance in men and to a different pharmacokinetics secondary to fat tissue abundancy in females [13, 14]. For the same reason, women present greater adverse effects on most anticancer therapies [15–18].

Despite growing evidence in other cancers, information on gender effect in MPNs is scarce. To understand whether and to what extent female sex could have an impact on PV biology and clinics, a detailed search of the literature was conducted using PubMed (US National Library of Medicine and the National Institutes of Health) and Web of Science (Thomas Reuters Online Academic Citation Index), with publication dates ranging from 1956 to March 2020. To ensure an extensive range of publications were identified, broad search terms for PV, sex, gender, and clinical/epidemiological variables (e.g., incidence, prevalence, frequency, diagnosis, pathogenesis, thrombosis, complications, survival, outcome) were utilized with the addition of alternative spellings and umbrella terms, e.g., erythremia and poliglobulia. Furthermore, we reviewed the literature cited in the identified papers.

Based on this research, we summarize the available data on the impact of female gender in light of the latest acquisitions on PV pathogenesis, diagnosis, epidemiology, clinical presentation, symptom burden, thrombotic risk, prognosis, and treatment strategies.

## Diagnostic criteria for PV are tailored on sex

In prepubescents, no sex difference is evident for hemoglobin (Hb) or hematocrit (HCT) levels [19]. On the contrary, during puberty, and in adults, a clear discrepancy emerges, as Hb level is on average 12% higher in males [19]. Androgens play a major role in ruling Hb value and plasma volume, even if the exact mechanism is not fully understood yet [20, 21]. Indeed, in adulthood testosterone is secreted in different concentrations between genders: levels are below 2 nmol/L in women, whereas its production rises to 20-fold and results 15-fold higher in men than in females [22]. Despite the latter are known to have reduced iron storages [23], sex effect on Hb/HCT seems to be independent from them [24, 25]. No interaction was also evident between gender and race [26] or circulating erythropoietin (Epo) levels [27].

Considering the physiological differences in Hb levels between genders, also conventional criteria for defining PV indicated higher threshold values in men (Table 1) [1, 28–35]. The updated World Health Organization (WHO) 2016 diagnostic criteria retained sex-based Hb levels but narrowed considerably the threshold between genders (Table 2) [1]. These changes were made to perform diagnosis earlier: recent studies have shown that patients with *JAK2* mutated thrombocytosis and moderately high HCT have increased thrombotic risk (“masked” PV), regardless of sex [36].

**Table 1:** Evolution of hemoglobin and hematocrit level thresholds by gender, through the latest diagnostic criteria for polycythemia vera

Diagnostic criteria, year	Hb levels cutoff (g/dL)	HCT levels cutoff (%)
BCSH, 1996 [28]		
M	n.r.	> 60
F	n.r.	> 56
WHO, 2001 [29]		
M	> 18.5	n.r.
F	> 16.5	n.r.
WHO, 2008 [30]		
M	> 18.5	> 99th percentile for age, sex
F	> 16.5	Altitude of residence
WHO, 2016 [1]		
M	> 16.5	> 49
F	> 16	> 48

*Hb*, hemoglobin; *HCT*, hematocrit; *M*, males; *F*, females; *BCSH*, British Committee for Standards in Hematology; *WHO*, World Health Organization. *n.r.*, not reported

**Table 2:** World Health Organization 2016 criteria for the diagnosis of polycythemia vera. A diagnosis of polycythemia vera requires either all three major criteria, or the first two major criteria plus the minor criterion

Major criteria	
Criterion no. 1, clinical	
Hemoglobin	> 16.5 g/dL in men, > 16.0 g/dL in women
Hematocrit	> 49% in men, > 48% in women
Red cell mass	25% increase above mean normal predicted value
Criterion no. 2, morphological	
Bone marrow morphology*	Hypercellularity for age with trilineage growth (panmyelosis), including prominent erythroid, granulocytic, and megakaryocytic proliferation with pleomorphic, mature megakaryocytes (differences in size)
Criterion no. 3, genetic	
JAK2 V617F mutation or JAK2 exon 12 mutation	Present
Minor criterion	
Serum erythropoietin level	Subnormal

*JAK2*, *Janus kinase 2*; *, major criterion no. 2 may not be required in cases with sustained absolute erythrocytosis: hemoglobin levels >18.5 g/ dL in men (hematocrit, 55.5%) or > 16.5 g/dL in women (hematocrit, 49.5%) if major criterion no. 3 and the minor criterion are present. However, initial myelofibrosis (present in up to 20% of patients) can be detected only with a bone marrow biopsy; this finding may predict a more rapid progression to secondary myelofibrosis

Together with Hb or HCT values, the presence of *JAK2* mutation and a compatible marrow histology establish nowadays PV diagnosis, even in the absence of reduced Epo levels [1]. Leukocytosis, thrombocytosis, and splenomegaly may provide useful clues. An important exception is PV patients being diagnosed because of splanchnic vein thrombosis (SVT) [37]. The latter are more frequently women and may present significant discrepancies between Hb and HCT levels, due to plasma volume expansion [37, 38].

## Incidence of PV is lower in females?

Among various registries, the incidence of PV ranges from 0.4 to 2.8 × 10^5^ per year [39]. Prevalence is 1/3300 people, considering the long life expectancy [39]. PV is a disease occurring mainly in the sixth decade [40] but can be diagnosed at any age. In some publications, it is reported that the risk of myeloid diseases is higher in young females, while in advanced ages it is overexpressed in males [41, 42]. More recently, 426 PV cases followed between 2001 and 2011 were extracted from the SEER (Surveillance, Epidemiology, and End Results) cancer registry [43]. Incidence rate increased significantly in patients aged over 50 years, and even more over 75 [43]. In all age groups, PV risk resulted lower among women [43]. These results parallel those from the ECLAP (European Collaboration on Low-dose Aspirin in Polycythemia Vera), the CYTO-PV (Cytoreductive therapy in PV) prospective studies [44, 45] (female rate was 40.5% and 36.8%, respectively), and a study on 70 young PV subjects (30%) [46]. Also, the subsequent SEER analysis from 2001 to 2016 showed a male to female incidence rate ratio of 1.6, which is consistent with data from European registries [39, 47]. These findings seem to indicate that the incidence rates of PV are lower in females, and this difference holds also in lower-age patients [43, 46].

In contrast to this statement, in two longitudinal studies collecting data on 165 Polycythemia Vera Study Group (PVSG)–defined and 3,382,008 WHO-defined patients, women represented 64% and 58% of the population, respectively [48, 49]. Finally, in a systematic meta-analysis, crude annual incidence did not significantly differ between males and females [3].

These epidemiological discrepancies may partially derive from subsequent changes in diagnostic criteria over a relatively short period of time. Also, MPNs are not always diagnosed in hospital settings and histopathological confirmation is not always performed. This might lead to an inaccurate case ascertainment by registries. On the other hand, claims-based analyses are prone to code errors and lack a validation process [50]. Additionally, the potential impact and changes over time of lifestyle habits on PV incidence may be relevant and requires further investigation. Very recently, MPN incidence was found to be closely related to smoking habits [51] which is generally more common in men.

Finally, selection bias may represent a major issue. For example, a highly different portion of females is enrolled in longitudinal studies (i.e., those collecting data from selected institutions or providing most prognostic information) compared with the fraction involved in interventional trials. A systematic comparison of patient characteristics in clinical vs longitudinal studies may demonstrate this bias’s relevance on the heterogeneity of results and on the quality of the final evidence.

## PV pathogenesis: A different genetic background between genders

*JAK2* mutation involves exon 14 (*JAK2*V617F, from now on *JAK2*) in 95–99% of PV patients and it is homozygous in around 30–50% of cases. The other 1–5% of subjects carry “gain of function” mutations in exon 12 [5, 52, 53]. The latter showed an association with younger age or higher Hb level, while no gender imbalance was identified in two independent cohorts [54, 55], suggesting that sex does not play a key role in exon 12 genetic instability.

At diagnosis, female PV patients present less frequently with a homozygous *JAK2* genotype compared with males (median: 61% vs 80%). This is consistent with a lower *JAK2* variant allele frequency (VAF) in the former, even adjusting for age and disease duration [56]. During follow-up, *JAK2* VAF is similar within the first year from diagnosis, but it becomes significantly higher in men (median: 82% vs 63%) when evaluated after at least eight years. A possible explanation is that a lower *JAK2* VAF induces fewer mitotic recombination events and less expansion of homozygous *JAK2* clones, leading to a predominant distribution within the PV phenotypes [48, 57]. A selective *JAK2* expression in platelets has been reported only in women with ET but not PV [56, 58].

In PV, *JAK2* mutation is not the only detectable genetic abnormality [59, 60]. Over 50% of patients showed non-driver sub-clonal aberrations in a next-generation sequencing (NGS) study [61]. Within 76 MPN patients sequenced at Johns Hopkins Hospital, females showed fewer additional somatic genetic alterations [62]. On the contrary, males present more frequently with non-driver mutations as *ASXL1*, *SRSF2*, *U2AF1*, *EZH2*, *IDH1*, and *IDH2* [62]. A Mayo Clinic-Florence collaboration analyzed 906 molecularly annotated MPNs, including 404 PV [53]. In this case, additional genetic abnormalities were found in about 18% of PV patients, without gender differences [53].

Of note, the *JAK2* mutation has been detected also in the general population with a frequency of 0.1–0.2% with increasing age and male sex associated with its acquisition. However, individuals who develop the *JAK2* mutation in the general population have a VAF below 2% and minimal evidence of MPN [63, 64].

Gene expression profile in circulating CD34+ cells from 19 PV cases has been investigated [65]. Authors found that fewer genes were differentially expressed in females than in males (235 vs 571), but the former had more than 3-fold activated molecular pathways. For instance, the pentose phosphate and the fatty acids pathways were not activated in males [65]. This finding suggests that selected metabolic mechanisms might play a role in the pathogenesis of PV in women [65]. In the same study, 102 genes with differential regulation were concordant between genders [65].

Concerning cytogenetic analysis in PV, around 20% had abnormalities, without sex differences [66, 67].

The inflammatory milieu plays an important role in MPNs. A field of active research is related to extracellular vesicles, which are small particles released by activated cells and involved in intercellular signaling. To date, no difference between men and women was found for the concentration of inflammatory biomarkers, such as high-sensitivity C-reactive protein or pentraxin-3 [68]. Consistently, in a cohort of 38 PV patients, no differences were observed in terms of circulating platelet-derived and tissue factor–positive extracellular vesicles (even though the content was increased in both genders) (Catani L, personal data). Table 3 summarizes peculiarities in epidemiology and the genetic background of PV in females.

**Table 3:** Epidemiology and molecular characteristics of polycythemia vera in women

Variable	Females (vs males)
Epidemiology	
Incidence rate	Lower in: – SEER registry 2001–2011 [43] – CYTO-PV and ECLAP prospective trials [44, 45] – Across any age group [46] – SEER registry 2001–2016 [49] – European registries review [39] Higher in: – previous diagnostic criteria [28, 32, 33] Comparable in: – systematic metanalysis [3]
Genetic background	
*JAK2*-mutated clone	– Less frequently homozygous [45] – Lower VAF at diagnosis and during long follow-up [56] – Selective expression in platelets [58]
Additional myeloid mutations by NGS	No difference in rate in the MIPSS-PV study [53]
Gene expression profile	Fewer genes differentially expressed, but more activated molecular pathways [65]
Cytogenetic abnormalities	No difference [66, 67]
Inflammatory mediators	No difference [68]

*SEER*, Surveillance, Epidemiology, and End Results; *CYTO-PV*, Cytoreductive therapy in Polycythemia Vera; *ECLAP*, European Collaboration on Low-dose Aspirin in Polycythemia Vera; *JAK2, Janus Kinase 2*; *VAF*, variant allele frequency; *NGS*, next-generation sequencing; *MIPSS-PV*, mutation-enhanced international prognostic scoring system for polycythemia vera

## PV phenotype in women: A milder clinical presentation with a relevant symptoms burden

The prospective ECLAP trial enrolled 1638 patients and therefore represented a valid tool to analyze clinical characteristics of PV [40]. Disease phenotype was milder in women compared with that in men: at study entry, the former showed lower rates of myocardial infarction (11.3% vs 5.8%) and peripheral arterial disease (6.1% vs 2.9%). This could partially depend on the reduced cardiovascular risk factors (i.e., smoking) incidence among females [69].

**Fig. 1:**
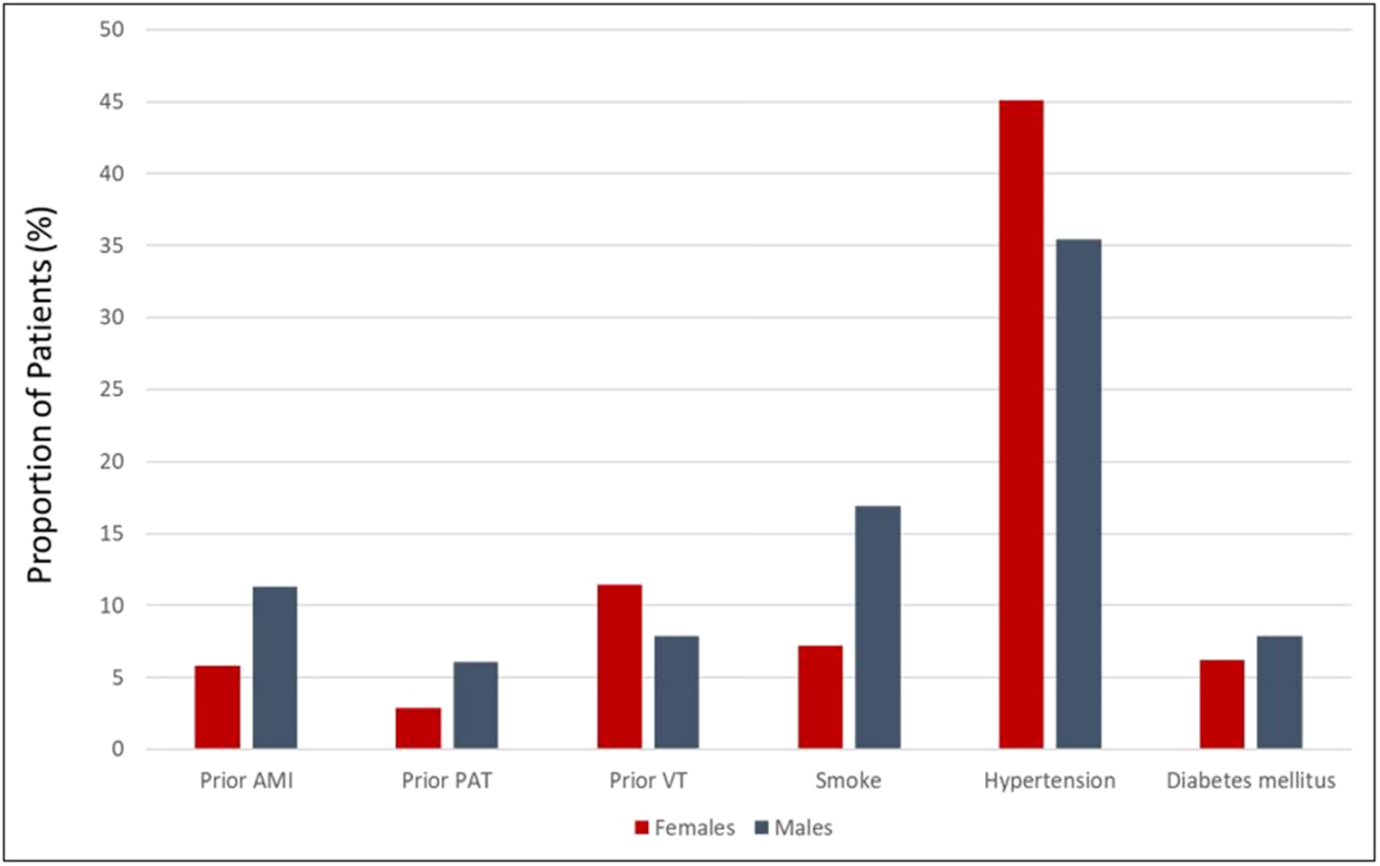
Thrombosis history and cardiovascular risk factors according to gender in a cohort of 1638 polycythemia vera patients (adapted by Landolfi et al. [40]). Legend: AMI, acute myocardial infarction; PAT, peripheral arterial thrombosis with residual intermittent claudication; VT, venous thrombosis

Besides, the ECLAP study underlined lower HCT levels (46 ± 6% vs 48 ± 6%) and smaller splenomegalies in women [40]. However, it should be acknowledged that the clinical phenotype of PV, particularly in terms of HCT levels, is significantly influenced by gender-specific diagnostic thresholds (Fig. 1).

Of note, a history of venous thrombosis was more common in females (11.4% vs 7.9%) [40] (Fig. 1). In particular, young women had a significantly increased rate of SVT, including extra-hepatic portal vein thrombosis and Budd-Chiari syndrome [38, 40, 69–72]. In a cohort of 270 consecutive *JAK*2-positive MPNs, 15 women had vascular complications at diagnosis: eight of them were affected by PV and had SVT [69]. Of note, this subgroup had lower leukocytosis, *JAK2* VAF, and cholesterol levels, along with less smoking history when compared with men [69]. However, mortality in patients with SVT does not seem to depend on gender [72]. A retrospective Spanish study suggested that the excess of fatality in cases of SVT was related to hepatic complications, major bleeding, and second cancers [72]. Recently, retrospective data on 518 MPN-related SVT cases (including 192 PV) have been reported [38]. Female patients were prevalent, reaching 53.1% in PV [38]. The median age at diagnosis was significantly lower in the case of SVT compared with MPNs controls [38]. The peculiar gender and age distribution in MPNs-SVT suggests that traditional risk factors for thrombosis are of limited contribution in this context [38]. The reasons for this distribution are not completely understood. Sex hormones probably play a pathogenic role. Additional risk factors for SVT in females may include the use of oral contraceptive or hormone replacement therapy in women [73–76]. Also, a hypercoagulable state could increase the risk of SVT [77, 78]. A strong association was reported between the presence of factor V Leiden and the development of Budd-Chiari syndrome or portal vein thrombosis (PVT), whereas prothrombin G20210A mutation was associated only with non-cirrhotic PVT [79].

Gender effect seems particularly important in determining symptoms burden. Out of 2006 MPNs patients that completed the Myeloproliferative Neoplasm-Symptom Assessment Form Total Symptom Score (MPN-SAF TSS) and the Brief Fatigue Inventory Patient Reported Outcome, females were 1089 (54.3%) [80]. The latter had more severe and frequent individual symptoms, along with overall higher TSS [80]. Looking at the symptoms list, those related to abdominal discomfort and microvascular disturbances (i.e., headache, dizziness) were predominant in women [80]. Notably, factors that may contribute to disease burden, like anemia or other adverse prognostic features, were comparable between genders [80]. However, there was not a corresponding decrease in the quality of life in females [80]. Whether this counterintuitive finding depends on the propensity of women to express their complaints, or to a greater capacity of men to tolerate symptoms, or by an overall low sensitivity of the MPN-SAF TSS remains to be clarified.

## Gender seems not predictive of thrombotic risk and treatment efficacy in PV

There is consistent evidence that thrombotic risk in PV is not significantly influenced by sex. Of note, in the ECLAP study, this holds true except for a higher incidence of SVT at or near diagnosis in female patients [40]. In the CYTO-PV study, only HCT levels and leukocyte count were significantly associated with increased risk of thrombosis, regardless of sex [45, 81]. The same incidence rate between genders was also found in a large cohort of MPNs prospectively evaluated at the Mayo Clinic, which included 711 PV patients [80]. Therefore, current PV therapeutic recommendations do not stratify vascular risk on sex, but according to age and thrombosis history [4, 82, 83]. Phlebotomies and aspirin are indicated in patients younger than 60 years of age, while cytoreduction is required in “high-risk” patients, that subject older than 60 years and/or with previous thrombosis [4, 82, 83]. Only recently, Karantanos et al. reported the outcome of 694 MPNs patients (394 females, 258 males; 311 ET, 252 PV, and 89 PMF) prospectively followed at the Johns Hopkins Hospital from 2005 to 2019 [62]. They observed that arterial ischemic events were more common in males, independently from driver mutation status and MPN subtype [62].

Regarding treatment [83], a major goal of PV therapy is keeping HCT levels below 45%, in order to reduce thrombotic complications [45]. Prospective trials such as the CYTO-PV study have definitively established that this HCT target is independent of gender [45, 84]. Some have advocated a lower HCT threshold (< 42%) as more appropriate in females [5, 84]. Among the reasons, the fact that, at every body weight, women normal red cell mass is around 600 mL lower than men [5, 84]. Therefore, when HCT is 45%, females have, at least, a blood excess of 600 mL [5, 84]. This difference could in part be explained by a 15-fold lower testosterone levels than men [22].

A recent consensus pointed out that a 40–42% HCT target is appropriate in subjects with persistent or recurrent symptoms of hyper-viscosity despite HCT < 45%, regardless of sex [70]. A stricter threshold seems not indicated even in case of pregnancy, at least in “low-risk” cases [85]. Despite the absence of formal proof, in a recent survey conducted among hematologist members of the MPN Research and Aplastic Anemia and Myelodisplastic Syndromes (MDS) Foundations, almost half reported to apply a gender-dependent approach for the maintenance of HCT in PV [86].

To date, scarce information is available on a possible different safety/efficacy profile of drug treatments in women with PV. In the CYTO-PV trial, 365 PV patients on therapy with phlebotomies, hydroxyurea (HU), or both were randomized to receive either more or less intensive treatment [45]. Reduction of cardiovascular events was higher in the former cohort and independent from sex, as well as age, previous thrombosis, platelet or leukocyte counts, splenomegaly, past cytoreductive treatment, or antiplatelet/anticoagulant drugs [45]. The observational study REVEAL prospectively followed 2510 PV patients in the USA [87]. The reported proportion of females receiving HU was comparable with that of males [87].

In a series of 890 PV cases from the Spanish Registry, Alvarez-Larran et al. investigated the clinical relevance of resistance/intolerance to HU, as defined by the 2009 European Leukemia Net (ELN) criteria [88, 89]. Resistance to HU was recorded in 5.7% of patients and distributed as follows: 3.3% need for phlebotomies, 1.6% uncontrolled myeloproliferation, 0.8% failure to reduce massive splenomegaly [89]. Intolerance to cytoreduction occurred in 10.7% of cases, mainly (9%) due to extra-hematological toxicity [89]. While older age correlated with resistance/intolerance to HU, female gender resulted significantly associated only with time to unacceptable extra-hematological toxicity [89]. Nevertheless, the latter did not increase mortality rate, risk of vascular complications, and of SMF/BP transformation [89].

Ruxolitinib is a *JAK1/JAK2* inhibitor that is used as a second-line therapy for PV patients’ resistant or intolerant to HU [90, 91]. Its approval was based on the results of the prospective clinical trials Response and Response-2, which enrolled 222 patients with and 173 without palpable splenomegaly, respectively [90, 91]. Subjects were randomized to receive either ruxolitinib or best available therapy (mainly HU) [90, 91]. The primary endpoint of the Response study was the proportion of patients achieving HCT control and at least a 35% reduction in spleen volume at week 32, while it was only HCT control at week 28 in Response-2 [90, 91]. In both trials, primary efficacy results were consistent across all subgroups, regardless of gender [90, 91]. Notably, also symptom control was comparable [90, 91]. Longer-term follow-up of these studies has not disclosed any significant sex difference in terms of response durability and toxicities [92, 93]. However, these studies had very heterogeneous designs (retrospective and prospective), inclusion criteria (high-risk patients with or without resistance or intolerance to HU), and primary endpoints (cardiovascular death or thrombotic event in the CYTO-PV study, rate of HCT/spleen control in the Response/Response-2 trials).

## The controversial role of female sex on PV survival

While a longer overall survival for female patients has been demonstrated in large cohorts of ET [53, 94, 95], the prognostic impact of gender in PV is still a matter of debate.

Many retrospective studies have demonstrated a clear survival disadvantage in patients with PV compared with age- and sex-matched healthy controls [7, 95–97]. The International Working Group for Myeloproliferative Neoplasms Research and Treatment (IWG-MRT) prognostic model for PV is the most used and it does not consider gender [97]. Indeed, this score divides patients into three risk categories based on the following demographic/laboratory characteristics: age, previous venous thrombosis, and leukocytosis [97]. Hazard ratio (HR)–weighted adverse points were assigned to age ≥ 67 years (5 points), age 57–66 years (2 points), leukocyte count ≥15 × 10^9^/L (1 point), and venous thrombosis (1 point), to devise a prognostic model that included low-risk (0 points), intermediate-risk (1–2 points), and high-risk (≥ 3 points) categories. The prognostic model stratified between high-risk (median survival = 8.3 years; HR, 11.1; 95% CI: 6.3–19.6), intermediate-risk (median survival = 15 years; HR, 3.5; 95% CI: 1.9–6.2), and low-risk (median survival = 26 years) patient groups [97]. In a retrospective cohort of 831 consecutive MPNs (396 PV and 435 ET), male sex was associated with shorter survival only in ET, but not in PV [95].

Conversely, in 261 PV retrospectively collected in the Spanish registry, men had a 2-fold increase in mortality rate compared with women (HR:2.3, 95% CI: 1.2–4.3, *p* = 0.008) [98]. Additionally, age > 65 years and leukocytosis >10 × 10^9^/L at diagnosis were associated with a significantly shorter survival [98]. Recently, the Mutation-enhanced International Prognostic Scoring System (MIPSS) was proposed for ET and PV [53]. Of note, male sex had an impact on ET survival (HR 1.8, 95% CI: 1.3–2.4), while it did not retain statistical significance in PV [53].

Evolution into PPV-MF occurs in around 5% and 15% of patients at 10 and 15 years of follow-up, respectively [95]. SMF transformation is associated with a significant worsening of symptoms and quality of life, along with a substantial reduction in survival expectation [2, 95, 99–101]. PV could also progress into BP in 3% and 10% of patients after 10 and 20 years from diagnosis, respectively [97]. This eventuality confers a very poor outcome, with a median survival of 1.5 to 2.5 months if not treated [102].

To date, sex is not included among risk factors for PV evolution into PPV-MF and BP. Conventional risk factors for PPV-MF and BP transformation include age ≥ 60 years, leukocytosis (≥ 15–30 × 10^9^/L), homozygosity for *JAK2* mutation, and exposures to alkylating agents (reviewed by Cuthbert D et al.) [103–110]. NGS based studies demonstrated also that non-driver mutations in myeloid genes could influence PV evolution: *ASXL1*, *SRSF2*, *RUNX1*, *SF3B1*, and *IDH1/2* for PPV-MF; *ASXL1*, *TP53*, *SRSF2*, *IDH1/2*, and *RUNX1* for BP [110]. In the recent MIPSS-ET/PV study, only 8 (2%) of PV patients showed “unfavorable” genetic alterations. *IDH2* and *RUNX1* mutations adversely affected BP-free survival (BP-FS) and *U2AF1* SMF-FS, when considering the Mayo Clinic cohort. As for Florence database, *SRSF2* influenced only BP-FS [53].

Looking at young PV patients, 70 subjects aged less than 50 years were followed for a median time of 14 years in a monocentric cohort [46]. Overall, five (7%) patients developed BP and the same percentage PPV-MF, with a 20-year cumulative risk of 15% and 10%, respectively [46]. Leukocytosis resulted in the only clinical parameter associated with PPV-MF evolution, while other variables as gender did not influence disease progression [46].

The MYSEC (Myelofibrosis Secondary to Polycythemia vera and Essential Thrombocytemia) project is an international collaboration that collected 684 molecularly annotated SMF [101, 111]. In this cohort, ET and PV diagnosis occurred at a younger age in females (median 50 vs 53 years) while age at the time of SMF transformation was similar between genders (median 63 vs 65 years) [111]. Therefore, ET and PV seem to evolve slower in females, as documented by a longer time to progression into SMF compared with men (median 11.3 vs 10.1 years) [111]. Whether this observation is linked to a different disease biology among females, or to a higher propensity of the latter to undergo blood tests and medical evaluation, is still to be determined.

Considering the whole MYSEC SMF dataset, women were characterized by a more indolent phenotype: higher platelet count, smaller splenomegaly, and lower circulating blasts [111]. Besides, they showed a longer life expectancy, with a median survival of 10.1 compared with 8.1 years in males [111]. Actually, looking only at the PPV-MF sub-cohort, gender did not impact neither the above clinical parameters nor mortality [111, 112].

**Fig. 2:**
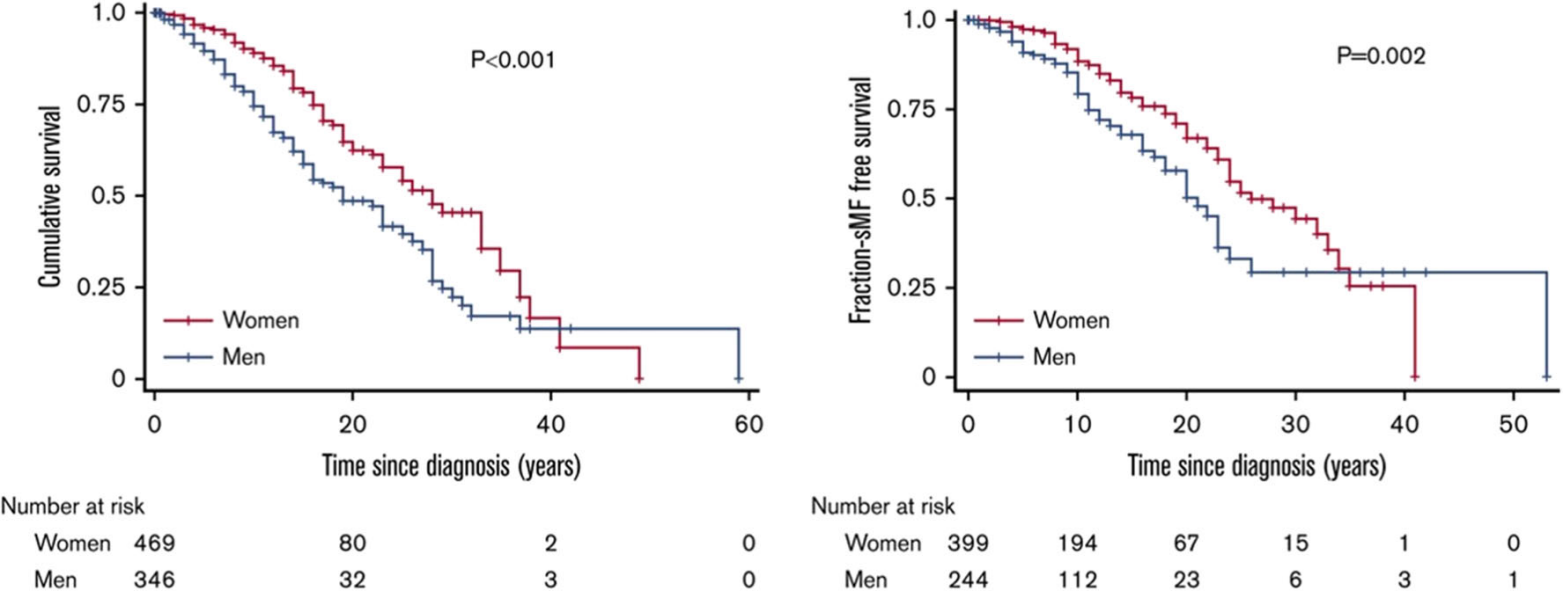
Men with MPNs have worse survival, and shorter time to MF transformation compared with females. Reproduced by Karantanos T et al. [62]

There are anyway some data advocating for a prognostic relevance of sex in PV [96]. In a sub-analysis of the ECLAP study, BP or MDS were diagnosed in 22 patients [96]. Notably, together with age older than 60 years and lower blood cholesterol levels, also female sex was strongly associated with increased risk of BP/MDS progression [96]. In Karantanos et al., male sex is predictive of SMF risk, independently from age, clinical phenotype at diagnosis, and driver mutation status [62]. Besides, the authors found a significant association with BP progression, independently from age at diagnosis and driver mutations [62] (Fig. 2).

Prognostic factors for mortality, evolution into PPV-MF, or into BP in female PV patients are listed in Table 4.

**Table 4:** Risk factors for clonal evolution and overall survival in female patients affected by polycythemia vera

Event	Female gender
Death	Conflicting data: – No impact as for the IWG-MRT [44] and MIPSS-PV (*p* = 0.7) models [53] – Lower risk in a retrospective cohort (*p* = 0.008) [45]
PPV-MF	No difference in the MYSEC as for [91]: – time to progression into PPV-MF (*p* = 0.49) – the phenotype at PPV-MF diagnosis – mortality (*p* = 0.45) Lower risk of evolution (Johns Hopkins Hospital) (*p* = 0.013) [62]
BP	Conflicting data: – Increased risk (ECLAP sub-analysis) (*p* = 0.04) [43] – Lower rate (Johns Hopkins Hospital) (*p* < 0.001) [62]

*IWG-MRT*, International Working Group for Myeloproliferative Neoplasms Research and Treatment; *MIPSS-PV*, Mutation-enhanced International Prognostic Scoring System for Polycythemia Vera; *MYSEC*, Myelofibrosis Secondary project; *PPV-MF*, post-polycythemia vera myelofibrosis; *MYSEC-PM*, MYSEC-prognostic model; *BP*, blast phase; *ECLAP*, European Collaboration on Low-dose Aspirin in Polycythemia Vera

## The unique aspects of polycythemia vera in women: Facing reproductive issues

Considering the abovementioned median age at diagnosis, around 15% of PV patients are less than 40 years of age at the time of diagnosis [41]. Therefore, women of childbearing age may suffer from PV, which can also be diagnosed in pregnancy or during investigations for recurrent pregnancy loss. To date, only case reports and small cohorts [113–115] are specifically focused on pregnancies in PV patients. More data are available in ET [116].

In 2005, Robinson et al. analyzed 18 pregnancies in 8 women affected by PV: about 65% had a positive outcome, while about 25% developed maternal complications [113]. A more recent Italian paper retrospectively collected 24 pregnancies in 15 PV cases and confirmed a comparable live birth rate (62.5%, of which 41.7% ended with a full term and 20.8% with a preterm delivery) [115]. The incidence of maternal complications was slightly lower (16.7%) [115] and nine (37.5%) pregnancies ended because of fetal loss, which was a late event in 44% cases [115]. In a subsequent series of 11 pregnancies in 7 females with PV, 8 (72.7%) ended with a full-term delivery [117]. In a recent meta-analysis including a total of 779 women and 1226 pregnancies in patients with PV and ET, the live birth rate was higher in MPN patients who received low-dose aspirin during pregnancy than those managed with observation alone (11 studies, 227 patients; unadjusted OR, 8.6; 95% CI, 4.0–18.1, *p* < 0.001). Also, a trend (albeit not statistically significant) towards further improvement in the live birth rate with the addition of low molecular weight heparin (LMWH) to aspirin was observed (6 studies, 96 pregnancies; unadjusted OR, 2.1 for aspirin with heparin vs aspirin alone; 95% CI, 0.5–9.0, *p* = 0.30). Finally, the use of IFN with or without aspirin or heparin during pregnancy was associated with a higher live birth rate (6 studies, 90 patients, unadjusted OR, 9.7; 95% CI, 2.3–41.0; *p* = 0.002) [116]. Despite the limited numbers, maternal morbidity and gestational outcome are similar to ET [116] and secondary to thrombotic events.

In pregnant ET, the role of *JAK*2 has been debatable, with studies suggesting its correlation with late fetal loss, and others denying this evidence [57, 115, 116, 118, 119]. Even if PV is strictly associated with *JAK2*, no association has yet been found between pregnancy complications and differential type of mutation (exon 12 vs exon 14) or mutation load (heterozygous vs homozygous).

Indications on gestation management in MPNs are mainly derived from experts’ consensus. The 2011 ELN treatment MPN guidelines state that a pregnancy must be considered at high risk for negative outcome if the patient presents at least one of the following PV-related complications: (1) venous/arterial thrombosis or bleeding (whether pregnant or not); (2) three recurrent first-trimester losses, intrauterine growth restriction, fetal death, stillbirth; (3) severe pre-eclampsia [120]. Besides, placental abruption, significant ante- or postpartum bleeding or platelet count greater than 1.500 × 10^9^/L are associated with increased risk of complications during pregnancy [120]. According to ELN guidelines, all PV cases should be managed with low-dose aspirin until delivery and phlebotomies in order to keep HCT below 45%, at least in low-risk pregnancies [85, 120]. A stricter HCT threshold has been recently advocated in high-risk patients [85, 120]. In addition, prophylactic dose low molecular weight heparin should be given after delivery, until 6 weeks postpartum [120]. In unfavorable cases, patient’s management depends on the type of risk: if there were major thrombosis or severe pregnancy complications, low molecular weight heparin throughout the gestation period should be added. In the case of extreme thrombocytosis or previous major bleeding, it is indicated to avoid low-dose aspirin and consider interferon alfa [120].

More detailed indications emerged from a more recent comprehensive review [121]. Of note, cytoreductive therapy is recommended if already indicated before pregnancy or in the case of a high-risk pregnancy. Although none of the cytoreductive drugs used in MPN have a product license for use in pregnancy, IFN is the drug of choice, since it was never associated with teratogenic effects [122–124]. Conversely, fetal abnormalities were reported in humans and animals exposed to HU [125, 126]. Anagrelide may cross the placenta and have the potential to cause fetal thrombocytopenia in pregnancy [127]. As for pre-conceptual planning, it is important to optimize MPN management, to correct cardiovascular risk factors, and counsel women about pregnancy complications. During the gestational period, fetal growth should be regularly monitored by ultrasound. Maternal dehydration and immobility must be avoided. Breast feeding should be planned on an individual basis, considering the treatment needs. Low-dose aspirin seems to be excreted in very small amount in human milk, and the daily use of an 81-mg dose of aspirin should be considered safe during lactation [128].

Finally, the use of combined oral contraceptive either as contraception or to control excessive menstrual bleeding is not appropriate in female PV patients due to risks of venous thrombosis [129]. Other forms of contraception such as the progesterone-only pill, implant, depot contraceptive injection, or intrauterine systems are acceptable [130].

## Conclusions

For decades, gender differences in PV were not considered an investigational priority, given the paucity of exploratory tools in this rare disease. In recent years, the need for efficient treatments has led to increased acquisitions in terms of PV pathogenesis, phenotype, and prognosis. In this contest, sex has emerged as a potential disease modifier, despite its role is still controversial under many aspects, including thrombotic risk, and survival. The mechanisms by which sex may influence disease course and its interactions with other environmental and cultural factors remain also to be clarified. However, this review underscores the importance of clinical and translational gender-based research. In the coming years, collecting prospective data on PV patients stratified by sex will probably contribute to the development of personalized treatment strategies.

## Data Availability

Not applicable.
